# Trends in television time, non-gaming PC use and moderate-to-vigorous physical activity among German adolescents 2002–2010

**DOI:** 10.1186/1471-2458-14-351

**Published:** 2014-04-12

**Authors:** Jens Bucksch, Joanna Inchley, Zdenek Hamrik, Emily Finne, Petra Kolip

**Affiliations:** 1 WHO Collaborating Centre for Child and Adolescent Health Promotion, Bielefeld University, School of Public Health, PO Box 100131, D-33501 Bielefeld, Germany; 2 Child and Adolescent Health Research Unit, School of Medicine, University of St Andrews, North Haugh, St Andrews Fife KY16 9TF, UK; 3 Faculty of Physical Culture, Department of Recreation and Leisure Studies, Palacký University Olomouc, Tr. Miru 115, 771 11 Olomouc, Czech Republic

**Keywords:** Television time, Non-gaming PC use, Moderate-to-vigorous physical activity, Trends, Germany, HBSC

## Abstract

**Background:**

Studies in youth highlight that moderate-to-vigorous physical activity (MVPA) and screen-time behaviours such as television viewing and PC use are associated with a range of health outcomes. However, little is known about recent trends in these behaviours in adolescents. This paper presents time trends in German adolescents’ television time, non-gaming PC use as well as MVPA from 2002 to 2010.

**Methods:**

Data were derived from the cross-sectional German Health Behaviour in School-aged Children (HBSC) study in 2002, 2006 and 2010. Analyses were based on 16,918 11-to 15-year olds boys (49.1%) and girls. Outcome variables were time spent in TV viewing and using a PC (weekday and weekend day) as well as the number of days achieving 60 minutes of MVPA. Changes in both screen-time behaviours and MVPA over time were analysed using sex-specific linear regression, controlling for age and family affluence.

**Results:**

TV viewing on weekdays, but not at weekends, declined steadily over time with a difference between 2002 and 2010 of 12.4 min/day in girls and 18.3 min/day in boys (p for trend < .01). We found a strong increase in PC use for non-gaming purposes over time for girls only, with a difference between 2002 and 2010 of 54.1 min/weekday and 68.8 min/weekend day (p < .001). For MVPA we found a slight statistically significant increase in terms of meeting PA guidelines as well as days/week in MVPA for boys and girls (p < .001). In 2010 14.0% of girls and 19.9% of boys met PA guideline.

**Conclusion:**

Although MVPA increased from 2002 to 2010 in German adolescents, the time spent in MVPA was still low. Despite the observed decrease in TV viewing, there was no overall decline in the observed screen-based behaviours, especially for girls. This is mainly due to a marked increase in use of a PC for chatting on-line, internet, emailing, homework etc. among girls during the last ten years which outweighs the corresponding decrease in TV viewing. The findings highlight a need for strategies and interventions aimed at reducing screen-time behaviours and promoting MVPA.

## Background

Numerous studies have demonstrated a strong association between moderate-to-vigorous physical activity (MVPA) and health in youth [1]. There is also a growing body of evidence highlighting that sedentary screen-time behaviours are linked to negative health outcomes in adults, including all-cause mortality independent of PA [2]. Systematic reviews in children and adolescents also underpin the association of screen-time behaviours with a range of physical (e.g. body composition) and psychosocial health outcomes (e.g. self-esteem, academic achievement) that are independent of PA levels [3,4]. Since PA and screen time moderately track from childhood to adulthood [5,6] and PA levels decline and screen time increase during adolescence [7,8], it is important to develop interventions to foster PA and to reduce screen time among children and adolescents. In addition, studies of adolescents have found a complex clustering of health behaviours, including PA and screen time, that should be considered in multimodal interventions to be more effective [9,10]. From a public health perspective it is also essential to monitor trends in health-related behaviours to provide information about the success of appropriate policies and programs as well as the need to develop and improve PA interventions.

Several reviews came to inconsistent conclusions about international trends in PA among adolescents. Two reviews indicated declining trends in PA especially in the domains of active transport, physical education and organized sports [11]. However, not all findings, especially those for vigorous intensity PA, were in line with a decline and, compared to adults, only a small number of studies were identified [12]. In contrast, a recent review of subjectively and objectively measured PA by Ekelund and colleagues [13] did not find a decline in PA over the last two decades. For both overall PA and sport participation no change or a slight increase was observed. However, few adolescents meet current PA guidelines that point to the health-enhancing effects of MVPA [14,15]. Trends of MVPA levels are therefore of special interest because of its relevance for health.

Little is known about recent trends in screen time behaviours. A review by Marshall et al. [16] reported that time budgets for total media use and TV viewing over the past five decades among adolescents have been fairly stable. Studies from different countries have shown that, during the last decade, time allocated to TV viewing has decreased but has been replaced by an increase in PC use (for both non-gaming and gaming purposes). Overall time spent in screen-based behaviours therefore remains about the same over this time frame [17,18]. However, there is no consistent pattern of time trends in sedentary behaviour across different studies [19-22].

The objective of the present analyses was to examine time trends in German adolescents’ television time and non-gaming PC use as well as MVPA. Since the findings on trends in MVPA and screen-time behaviours are inconsistent, we aim to clarify for German boys and girls aged 11 to 15 years the direction (stable, increase, decrease) of trends over a time frame of 8 years from 2002 to 2010.

## Methods

The Health Behaviour in School-aged Children (HBSC) study is a cross-national survey of school students that collects data every four years on 11-, 13- and 15-year-old adolescents. The scope of the survey covers a broad range of self-reported health behaviours and health outcomes among adolescents. This paper presents data from the past three German HBSC surveys. Each was conducted in accordance with the study protocol prepared by the HBSC International Coordinating Centre [23].

### Sample

The current analyses are based on the German HBSC trendfile. The compilation of this data set is described in detail elsewhere [24]. In brief, the German HBSC trendfile includes the surveys conducted in 2002, 2006 and 2010. Each of the three samples was based on a random sampling procedure of schools stratified by federal state, administrative district and type of school. In accordance with the international protocol only 11-, 13- and 15-year-old adolescents were surveyed by self-reported questionnaire. In total 17,929 (2002: n = 5,650, 2006: n = 7,274, 2010: n = 5,005) pupils were recruited. The school response rate varied between surveys from 39-48% and the pupil response rate in all three waves was >80%. HBSC surveys have been carried out in Germany since 1993/94. The first two surveys were limited to one out of 16 federal states. In 2002 and 2006 HBSC data were collected in 4 and 5 federal states respectively (representing geographically different parts of Germany) and combined to a nationwide German sample. The latest survey was nationally representative for German public schools. Because the first two surveys were regional in nature and the variables of interest have been partially introduced in 2002, they have been omitted from the trendfile. The sample was slightly reduced due to missing values in physical activity, sedentary behaviours as well as covariates (ranging from 5.0% missing values for weekday PC use to 7.3% for weekend TV viewing).

### Survey items

#### Moderate-to-vigorous intense PA

MVPA was assessed by asking: “On how many days in the past week were you physically active for 60 minutes or more”. Physical activity was defined as “any activity that increases your heart rate and makes you get out of breath some of the time” with examples of such activities. Response categories were: “0 days”, “1”, “2”, etc. up to “7 days”. This question was originally developed by Prochaska et al. [25] and also asked for physically active days in a typical week. For this original version the test-retest stability (ICC = .77; ICC = .82) [25,26] and validity in terms of substantial correlations with accelerometers was shown (r = .40; r = .49) [25,27]. Due to the limited space in the HBSC survey and high correlation between activity in the “past 7 days” and “a typical week”, only the “past week” item was used in 2006 and 2010. Consequently only this item is available for the trend analyses.

We used two variables to examine trends in MVPA. First the variable was interpreted as a continuous outcome variable. Second, in accordance with current PA guidelines [1], we created a dichotomous outcome variable. Those adolescents responding that they were active for at least 60 minutes on each of the last seven days were classified as meeting current guidelines, and vice versa.

### Screen time

Two screen time behaviours have been consistently reported in the last three German HBSC surveys. The first one asks “About how many hours a day do you usually watch television (including DVDs and videos) in your free time? “The DVD part was added in 2006. The second one asks “About how many hours a day do you usually use a computer for chatting on-line, internet, emailing, homework etc. in your free time?”. This item was asked in a more general way in 2002 and included use of a computer for gaming in a very generic way. We do not report on trends for computer/console games as a separate and differentiated item on gaming has only been included in HBSC since 2006. Nine possible response categories are given ranging from “none at all” up to more than 7 hours/day. Each item is subdivided to a weekday and a weekend day part. We interpreted the items as continuous and therefore individual answers were first recoded: into “None at all” = 0, “About half an hour a day” = .5, “About 1 hour a day” = 1, “About 2 hours a day” = 2 etc. and “more than 7 hours a day” = 7.5. To present minutes a day the recoded value was multiplied by 60. Test-retest reliability was assessed previously and found to be acceptable [28,29].

### Confounders

Since physical activity and screen time differ between boys and girls [7,8] all analyses were sex specific. Age and family affluence were considered important correlates of PA [30] and were controlled for in the regression analyses. Since the sampling was based on three specific age groups (i.e. 11-, 13- and 15- year olds), age was treated as a three-stage categorical variable. The family affluence scale was used as a proxy of socio-economic status. This scale consists of four items measuring material affluence that are self-reported by adolescents themselves. The items have been selected to characterize a child’s household by asking about car ownership (0, 1, 2 or more), computer ownership (0, 1, 2, 3 or more), number of family holidays last year (0, 1, 2, 3 or more), and own bedroom (no = 0, yes = 1). To derive a composite family affluence score we summed up the responses of the items. The family affluence scale has been validated against parent’s reports and national wealth indicators such as the Gross Domestic Product indicating acceptable validity [31]. The composite sum score was treated as a continuous variable. Where there was a significant interaction of survey year with family affluence, we used established cut-points to create low, middle and high affluent groups [31] to analyse group specific time trends.

### Data analysis

Analyses were conducted with SPSS v19, using the complex samples module to account for the clustered study design with “school class” as primary sampling unit. Since in 2002 the variable class was not coded we used instead the person’s identification. To correct for the lack of the cluster variable the sample was down-weighted by a factor of 1.2, the standard sampling design effect of the HBSC [32]. Descriptive data are presented as mean and standard deviations for each survey year. In addition, we reported the absolute difference in minutes per week (each screen time for weekend day and weekday separately) or in days per week with 60 min of MVPA. The significance of a linear trend was tested by treating survey year as categorical in regression analyses, controlling for age group and family affluence.

Changes in time spent in MVPA, TV viewing and (non-gaming) computer use across survey years were analysed using multiple linear regression adjusted for age group and family affluence, with survey 2002 as the reference year. The beta coefficient represents the change in minutes per day of screen time or in days per week of MVPA in 2010 or 2006 compared to 2002 (a positive coefficient indicates an increase over time). In addition we examined interactions of we reported with time (survey year) to determine if trends varied across subgroups. In the case of meeting current physical activity guidelines, we describe changes in proportions and used multiple logistic regression to compute changes over time in the same manner as described for linear regression. Results are presented separately for boys and girls. The level of significance was 0.05.

## Results

Sample characteristics are shown in Table 1. Table 2 shows, for both girls and boys, the means and the absolute difference in days per week of MVPA as well as the proportion meeting 60 min MVPA on each day of the week and the mean number of minutes per day in screen time behaviours over time. For MVPA, there is a slight increase in both outcome variables over time and this trend is similar for boys and girls. The absolute difference in MVPA days per week from 2002 to 2010 is +0.48 in girls and +0.39 in boys. The p for trend is significant. Looking at screen time, a more complex picture is evident. TV viewing on weekdays, but not at weekends, declined steadily over time with a difference of 12.4 min/day in girls and 18.3 min/day in boys (p for trend < .01). Non-gaming PC use increased significantly over time for girls only, with a difference of 54.1 min/weekday and 68.8 min/weekend day (p < .001).

**Table 1:** Study characteristics of the three surveys

	**2002**		**2006**		**2010**		**All**	
	**N**	**%**	**N**	**%**	**N**	**%**	**N**	**%**
**Gender**								
Girls	2864	50.7	3606	49.6	2576	51.5	9046	50.5
Boys	2786	49.3	3668	50.4	2429	48.5	8883	49.5
**Age**								
11	2094	37.2	2231	30.9	1687	34.0	6012	33.7
13	1800	31.9	2441	33,8	1628	32.9	5869	32.9
15	1741	30.9	2552	35.3	1640	33.1	5933	33.3

**Table 2:** Changes (means [95%-Confidence Interval]) and absolute differences in days of MVPA and minutes spent in television viewing and non-gaming PC use between 2002 and 2010

	**Girls/Mean [95% CI]**				**Boys/Mean**			
	**Survey year 2002**	**Survey year 2006**	**Survey year 2010**	**Difference**^**a**^**/p for trend**^**b**^	**Survey year 2001/2002**	**Survey year 2005/2006**	**Survey year 2009/2010**	**Difference**^**a**^**/p-for trend**^**b**^
**MVPA (days/week)**	3.43 [3.36-3.50]	3.79 [3.71-3.86]	3.91 [3.82-4.00]	+0.48/**p < .001**	3.96 [3.89-4.04]	4.29 [4.22-4.36]	4.35 [4.26-4.44]	+0.39/ **p < .001**
**% of meeting PA Guideline**	8.6 [7.6-9.7]	13.9 [12.6-15.3]	14.0 [12.5-15.5]	+5.4/**p < .001**	14.8 [13.5-16.3]	19.8 [18.3-21.4]	19.9 [18.0-21.9]	+5.1/**p < .001**
**TV (min/weekday)**	133.3 [129.8-136.8]	128.2 [123.8-132.7]	120.9 [116.2-125.6]	-12.4/ **p < .01**	145.3 [141.6-149.0]	138.1 [133.6-142.6]	127.0 [122.0-131.9]	-18.3/ **p < .001**
**TV (min/weekend day)**	180.7 [176.6-184.8]	189.5 [184.6-194.4]	180.7 [175.3-186.1]	+ - 0/ p > .05	205.6 [201.1-210.1]	210.3 [205.1-215.5]	201.6 [195.5-207.7]	-4.0/ p > .05
**PC use (min/weekday)**	42.1 [39.9-44.2]	78.2 [73.8-82.6]	96.2 [90.6-101.8]	+54.1/ **p < .001**	88.8 [85.3-92.3]	84.6 [79.8-89.3]	93.4 [87.5-99.4]	+4.6 / p > .05
**PC use (min/weekend day)**	58.9 [56.1-61.6]	100.8 [95.4-106.1]	128.7 [121.9-135.6]	+68.8 / **p < .001**	124.7 [120.3-129.2]	111.1 [105.1-117.1]	128.5 [121.3-135.6]	+3.8 / p > .05

Significant p for trends are in bold fonts.

^a^:Difference between 2010 and 2002 in days/week, min/day, percentages of meeting PA guideline.

^b^:p for trend is controlled for family affluence and age group.

Tables 3 and 4 show the beta values for the change in MVPA and screen time behaviours over time compared to the reference survey of 2002, controlling for age and family affluence. The findings confirm the univariate means from Table 2. Taking the covariates separately into account we observed a strong significant association with age in both girls and boys such that older adolescents have lower MVPA levels, watch more TV and use the PC more often.

**Table 3:** Change in regression coefficients for MVPA, television viewing and non-gaming PC use screen time behaviours over time compared to 2002 in girls adjusting for covariates

**Girls**	**MVPA (beta, SE, p-value)**	**PA guideline**	**TV min per weekday**	**TV min per weekend day**	**PC min per weekday**	**PC min per weekend day**
**Survey year**						
2002	Reference	Reference	Reference	Reference	Reference	Reference
2006	**.484, 0.92, <.001**	**.571, .095, <.001**	-.082, .044, >.05	**.669, .173, <.001**	.146, .113, >.05	.200, .138, >.05
2010	**.611, .093, <.001**	**.584, .089, <.001**	**.138, .047, <.01**	**.468, .201, <.05**	**.774, .172, <.001**	**1.209, .204, <.001**
**FAS**^**a**^	**.154, .014, <.001**	.024, .025, >.05	**-.161, .013, <.001**	**-.088, .023, <.001**	**.057, .012, <.001**	**.096, .015, <.001**
**AGE**						
11	Reference	Reference	Reference	Reference	Reference	Reference
13	**-.219, .084, <.01**	**-.456, .085, <.001**	**.583, .049, <.001**	**.700, .055, <.001**	**.017, .045, <.001**	**.245, .055, <.001**
15	**-.433, .085, <.001**	**-.825, .084, <.001**	**.629, 046, <.001**	**.665, .056, <.001**	**.086, .04, <.05**	**.184, .055, <.001**
**Survey year X FAS**^**a**^	n.s.	n.s.	n.s.	**<.05**	**<.05**	**<.05**
**Survey year X Age**	Significant	n.s.	n.s	n.s.	**<.05**	**<.05**

Significant findings in bold fonts; ^a^ = Family Affluence Scale.

**Table 4:** Change in regression coefficients for MVPA, television viewing and non-gaming PC use screen time behaviours over time compared to 2002 in boys adjusting for covariates

**Boys**	**MVPA (beta, SE, p-value)**	**PA guideline**	**TV min per weekday**	**TV min per weekend day**	**PC min per weekday**	**PC min per weekend day**
**Survey year**	Sig.					
2002	Reference	Reference	Reference	Reference	Reference	Reference
2006	**.382, .09, <.001**	**.330, .075, <.001**	**-.118, .047, <.05**	.064, .056, >.05	-.014, .166, >.05	.065, .199, >.05
2010	**.51, ,10, <0.001**	**.376, .082, <.001**	**-.240, .049, <.001**	-.017, .062, >.05	**.547, .208, <.01**	**.889, .247, <.001**
**FAS**^**a**^	**.087, .016, <.001**	-.022, .022, >.05	**-.136, .014, <.001**	**-.119, .018, <.001**	**.108, .019, <.001**	**.172, .024, <.001**
**AGE**						
11	Reference	Reference	Reference	Reference	Reference	Reference
13	-.138, .091, >.05	**-.314, .076, <.001**	**.511, .050, <.001**	**.638, .060, <.001**	**.530, .066, <.001**	**.798, .083, <.001**
15	**-.253, 0,92 < .01**	**-.589, .076, <.001**	**.561, .051, <.001**	**.659, .063, <.001**	**.698, .072, <.001**	**1,029, .096, <.001**
**Survey year X FAS**^**a**^	n.s.	n.s.	n.s.	n.s.	**<.05**	**<.05**
**Survey year X Age**	**<.05**	n.s.	n.s	n.s.	**<.05**	**<.05**

Significant findings in bold fonts; ^a^ = Family Affluence Scale.

We also examined survey year x family affluence and survey year x age group interactions (see Tables 3 and 4). In both girls and boys we found a significant survey year x age interaction for days of MVPA. Calculating age-specific models (data not shown) we identified a similar pattern for boys and girls explaining this interaction; a continuous increase in MVPA is observed among 11- and 13-year olds only. For 15-year-olds the main increase in MVPA is from 2002 to 2006. Interactions for watching TV were only significant for survey year x family affluence in girls. This is explained by the fact that the decrease is stronger among middle and lower affluent girls. In relation to PC use, significant interactions were observed for both age and family affluence with survey year. The patterns of interaction differed between boys and girls. Among girls, PC use (both weekday and weekend) increased in all age groups but the sharpest increase was among 15-year olds. Among boys, there was a decrease among 11-year olds and a clear increase was only evident among 15-year olds. The interaction with family affluence was even more complex. In girls, an increase in PC use for non-gaming purposes was observed for all family affluence groups. However, for weekday use the sharpest increase was in the lower affluent adolescents and for weekends we only found a slight increase among the highest affluent adolescents. In boys there was no clear pattern of interactions.

## Discussion

Although MVPA increased from 2002 to 2010 in German adolescents, the time spent in MVPA was still low. A slight decrease in TV viewing was observed for both girls and boys. This decrease was significant for weekdays only. However, there was no overall decline when taking into account both TV viewing and non-gaming PC use, especially for girls. Using PC for chatting on-line, internet, emailing, homework etc. in their free time has increased markedly in the last eight years in girls and outweighs the decrease in TV viewing. The interaction of time x age as well as time x family affluence with MVPA, TV viewing and non-gaming PC use generally showed that the degree of increase over time varied but there was no variation in the direction of change. The findings highlight a clear need for strategies and interventions aimed at reducing screen time and promoting MVPA levels. Although evidence shows that screen time behaviours and MVPA are distinct behaviours and only marginally correlated with each other, clustering of these two behaviours along with diet and other health behaviours have been reported in the literature. These studies suggest that it might be more effective to address all these behaviours together to make use of concepts like transfer learning [10,33].

Despite the observed increase in MVPA levels, only 14.0% of 11- to 15-year old girls and 19.9% of their male counterparts met recent PA guidelines. This prevalence is as low as in other German studies [34] as well as in many other countries [14,15] and is also confirmed by objectively measured MVPA levels [35]. Our findings on secular trends in behaviour are also confirmed by recent studies. The nationally representative Youth Risk Behavior Surveillance Surveys from the US found stable patterns in adolescents for MVPA from 1999 to 2005 [36]. In another U.S. sample of children aged 9–13 years, a slight increase in MVPA from 2002–2006 was found [22]. In contrast, two recent Czech studies observed declining daily steps and declining MVPA measured by self-report between 2000 and 2010 [17] as well as a stagnation or decline in the proportion of adolescents meeting MVPA guidelines [37]. Further trend studies in the context of the international HBSC study have been examined with indications of slight increases in vigorous intensity PA over time [38,39]. Taking recent studies and reviews into account the findings are quite inconsistent and depend on measurement issues as well as the domain, type or intensity of PA [12,13].

Our findings on age related declines in MVPA levels and insignificant associations between family affluence and meeting MVPA guidelines are in line with other German studies [34,40]. Our finding is not unique and is backed by other studies which also highlight that characteristics associated with social position of adolescents are not strong predictors of PA levels [41]. An explanation for our findings is that MVPA includes not only organized and vigorous intensity activities that depend on the availability of specific facilities or a club membership referring to a higher socio-economic status. A large amount of MVPA is based on activities of daily life (e.g. active commuting) or informal activities in parks etc. which may be less differentiated by socio-economic status [42]. It is important to take the domain of PA into account when examining the relationship between socio-economic status and PA levels [43].

In our study, we found that time spent watching TV decreased during the last ten years on weekdays for boys and girls. In contrast, non-gaming PC use increased markedly among girls, both for weekdays and weekend days, so that overall screen time was higher in 2010 compared to 2006 and 2002. For boys no statistically significant change was observed during this time period. A few other studies reported on screen time trends over time. One recent Czech study found a global increase in time allocated to different sedentary behaviours from 1998 to 2010 among girls. Interestingly, a decrease in TV viewing was replaced by an increase in PC use in both genders. Among girls, this increase was steeper than in our study [17]. Similarly, a study of Brazilian adolescents also reported a declining trend in TV viewing over the last decade that has been offset by computer use (non-gaming and gaming) in adolescent boys and girls [18]. Overall, however, we do not observe a consistent pattern of trends in TV viewing. A few studies have shown an increase in TV viewing or overall screen time [19,44,45] whereas other studies have found more stable or decreasing prevalence in TV viewing over time [21,38,46]. Two of the studies with increasing prevalence came from Chinese samples and might be not directly comparable to our cultural and secular context in terms of using screen-based media.

As supported by different studies [17,18,21,37] an increase in PC use is evident. One reason why we do not find an increase in non-gaming PC use in boys might be traced back to the fact that item PC use was limited to social and academic computer activities and did not include playing computer/console games. 2010 data from the national German HBSC survey found that boys spent more time playing computer games than girls [47] and therefore we might have also found increasing trends in overall PC use among boys if we had included this item in all three past surveys. For the HBSC wave in 2006 and 2010 for (data not shown) we found 110 minutes per weekday and 102 minutes per weekday on computer/console gaming in boys, respectively. However the item on “gaming” was not available for trend analyses from 2002 so that firm conclusions are not possible. Other studies highlighting increases in computer use over time often include both aspects of use [21]. The rapid increase in girls’s non-gaming computer use is an important finding likely to reflect both technological advances and socio-cultural shifts in peer communication during the last decade. One explanation might be the special interest of girls in the use of social media. Indeed, recent German data has shown that adolescents girls are more likely to use the internet for communication purposes (e-mails, chatting and online-communities) than boys [48]. In general, the use of internet increased in both genders in the last fifteen years [49].

Marshall and colleagues [16] reviewed the literature of the past 50 years and found no change in the time allocated to total media use (about 35–40 hours per week) and TV viewing (about 2.5 hours per day) among 11–17 year olds. In addition, the authors emphasize that the availability of TV in home settings and in bedrooms of children has increased enormously within this period so that from a public health perspective currently more young people are exposed to TV viewing. This finding is a further concern because having a TV in one’s own bedroom was identified as a strong correlate of TV viewing [8] that has also been associated with obesity [50]. However, although overall time spent using different media may not have changed, it is highly likely that the types of media and the time allocated to each type might have changed in recent years [16], as highlighted by our findings. With the continuous technological advances the choice of innovative and attractive devices (e.g. smartphones, tablets) supports high levels of screen time and makes comparisons over time difficult. In addition the simultaneous use of different media at one point in time is nowadays common [51], making it more and more difficult to disentangle the allocation of time to each different type of media. Overall, it is likely that the time using screens has been shifted away from television and traditional PC use and is underestimated by our study. In Germany, for example, the proportion of families having a tablet PC doubled from 2012 to 2013 [48] and the percentage of adolescents who own a smartphone increased from 0% in 2009 to 72% in 2013 [49]. However, this shift has mainly been taken place after our investigation period and might be not a concern for our findings. Future studies on screen time should be aware of this shift and have to differentiate the versatile nature of screen-time behaviour. The development of reliable and valid assessment tools is an important prerequisite in this context.

Our findings showed that TV viewing and non-gaming PC use increased with age. With regard to family affluence, we found that boys and girls from more affluent families have lower levels of TV consumption and higher levels of PC use. A recent review of correlates of sedentary behaviour confirmed age related increases in screen-based activities. It is also emphasized that indicators of lower socioeconomic status are associated with more screen-time but there are also studies showing that academic sedentary behaviours are more common among adolescents with higher socioeconomic status [8].

Finally, we found gender differences in the interaction of survey year x age as well as family affluence, especially for PC use. These interactions highlight target groups with the highest need for interventions. In terms of age, the findings suggest that 15-year old girls are a high priority group as they showed the sharpest increases in PC use especially for non-gaming purposes over time. However, we have to keep in mind that boys are more likely to use PC for gaming which was not included in the current study. This probably results in even higher levels of overall screen time. We therefore also have to consider the kind of screen time targeted in gender sensitive interventions. In addition, the family affluence interactions indicated greater increases in PC use among adolescents from low affluence groups compared with those from high affluence groups. This results in nearly equal minutes of PC use in girls in 2010. One explanation for this might be that in the last decade PC use has shifted away from academic to more social purposes leading to higher participation among all groups of socioeconomic background. An alternative explanation may be that computers have become increasingly affordable over the past decade. In boys the interactions are even harder to interpret, since PC use was relatively stable in the years from 2002 to 2010. More research on interaction is necessary to understand and confirm these findings.

Our data and findings have several limitations that should be kept in mind. First of all we used self-reported measures of MVPA and screen time and these are prone to misclassification, although previous studies have reported an acceptable reliability and validity for these measures [27,29]. Second, as we presented time trends some variations in prevalence over time might be attributable to changes in wording of the outcome variables. The item on TV viewing was revised in 2006 in terms of adding DVDs as one source of TV watching. With respect to the PC use item, the list of examples changed over time. In 2002, the question did not list “homework” but did include generic use of the computer for playing games. In 2006 and 2010 the list was enlarged by adding “for homework” and the example of playing games was deleted. The MVPA item was even slightly changed in wording. In 2002 the item was phrased “physically active or doing sports for 60 minutes or more”. In subsequent surveys “or doing sports” was deleted. Third, we only focused on two screen time behaviours. We did not report time trends for playing games on a computer or games console because this differentiated information has only been used since 2006 (see below). However the HBSC 2010 wave showed that this screen behaviour is also highly prevalent especially in boys [14,47]. An inclusion of gaming might have pointed to a stronger increase in screen time in boys. To understand and describe overall screen time it is important to examine all different types and domains. In addition, overall time being sedentary is even more complex to assess and screen time is only a weak proxy for it [52]. Finally, we note that our findings report cross-sectional trends and may not reflect longitudinal trends over time.

## Conclusion

In summary we found a slight increase in MVPA levels in the last decade but they were still low as reported in many other studies [15]. Therefore the results show that low levels of PA have persisted over the last decade and further investment is needed to improve levels of participation among the adolescent population. Promoting PA at a population level is a top priority for public health intervention in Germany since past and existing initiatives were not able to provoke substantial changes. With respect to screen time, our findings indicate increasing levels in the last decade. The slight decline in TV viewing is promising, because television time but not computer time is associated with unfavourable health behaviours such as snacking and exposure to food advertisements [53,54]. Concurrently, more and more studies point out that, on the one hand, the association between screen time and MVPA [55] is relatively independent and on the other hand the health risk of screen time is independent of MVPA levels in youth [3,4]. This has important implications for the current and future health of young people and it is essential that steps are taken to develop and disseminate interventions that reduce overall screen time from a sitting-health perspective [56]. However, it is important to recognize that screen time is not a homogenous behaviour and that different types of screen time may be associated with different health-related outcomes. For example computer use is related to the quality of peer relationship [57] and using electronic media is positively associated with the time spent together with friends [58].

## Abbreviations

MVPA: Moderate to vigorous physical activity; HBSC: Health behaviour in school-aged children - study.

## Competing interests

The authors declare that they have no competing interests.

## Authors’ contributions

JB and JI developed the study idea. JB drafted the first version of the manuscript and performed the statistical analyses in collaboration with EF. PK supervised the research process and critically reviewed the final version of the manuscript. JI, ZH, EF and PK supported the data interpretation and revised the draft of the manuscript. All authors read and approved the final version of the manuscript.

## Pre-publication history

The pre-publication history for this paper can be accessed here:

http://www.biomedcentral.com/1471-2458/14/351/prepub
